# PERCEPTIONS OF AGING: INVESTIGATING GROUP DIFFERENCES AND PREDICTORS OF SUBJECTIVE AGE

**DOI:** 10.1093/geroni/igae098.3277

**Published:** 2024-12-31

**Authors:** Grace da Rosa, Peter Martin

**Affiliations:** Georgia State University, Atlanta, Georgia, United States; Iowa State University, Ames, Iowa, United States

## Abstract

While aging is a universal process, individuals perceive and experience aging in markedly different ways. Subjective age reflects how people feel (younger or older) compared to their actual age. The purpose of this study was to assess the association of subjective age (younger vs. older) with education, actual age, self-report health, personality, loneliness, chronic stress, and psychological well-being. This study used data from 2000 wave of the Health and Retirement Study. A total of 3,113 adults 65-years and older were included in this study. The results revealed that older adults who feel younger compared to their chronological age reported lower depression scores, lower Neuroticism, lower levels of loneliness, and lower chronic stress, compared to individuals who perceived themselves as older than their chronological age. In addition, older adults who felt younger compared to their chronological age reported higher levels of Extraversion, Agreeableness, Conscientiousness, Openness to Experience, Life Satisfaction, Control over their health, Control over their social life, and Control over their financial situation. Significant linear regression results confirmed that high levels of Neuroticism, low Agreeableness, and low levels of hopefulness were associated with older perceived age. Higher levels of Extraversion and Openness to Experience and reported higher Control over health were associated with younger perceived age. In summary, this study offers valuable insights on the importance of subjective age. Interventions that target perceptions of aging may have positive effects on health behaviors, psychological functioning, and overall quality of life of older adults.

